# In vitro effect photodynamic therapy with differents photosensitizers on cariogenic microorganisms

**DOI:** 10.1186/s12866-015-0524-3

**Published:** 2015-09-26

**Authors:** P. Soria-Lozano, Y. Gilaberte, MP Paz-Cristobal, L. Pérez-Artiaga, V. Lampaya-Pérez, J. Aporta, V. Pérez-Laguna, I. García-Luque, MJ Revillo, A. Rezusta

**Affiliations:** Department of Microbiology, Hospital Universitario Miguel Servet, Zaragoza, Spain; Department of Dermatology, Hospital San Jorge, Huesca, Spain; Health Science Institute of Aragón, Zaragoza, Spain; Department of Applied Physics. Faculty of Science, University of Zaragoza, Zaragoza, Spain; Department of Microbiology, University of Sevilla, Sevilla, Spain; Department of Microbiology, University of Zaragoza, Zaragoza, Spain

## Abstract

**Background:**

Antimicrobial photodynamic therapy has been proposed as an alternative to suppress subgingival species. This results from the balance among *Streptococcus sanguis*, *Streptococcus mutans* and C*andida albicans* in the dental biofilm. Not all the photosensitizers have the same photodynamic effect against the different microorganims. The objective of this study is to compare in vitro the photodynamic effect of methylene blue (MB), rose Bengal (RB) and curcumin (CUR) in combination with white light on the cariogenic microorganism *S. mutans, S. sanguis* and *C. albicans.*

**Results:**

Photodynamic therapy with MB, RB and CUR inhibited 6 log 10 the growth of both bacteria but at different concentrations: 0.31–0.62 μg/ml and 0.62–1.25 μg/ml RB were needed to photoinactivate *S. mutans* and *S. sanguis*, respectively; 1.25–2.5 μg/ml MB for both species; whereas higher CUR concentrations (80–160 μg/ml and 160–320 μg/ml) were required to obtain the same reduction in *S. mutans* and *S. sanguis* viability respectively. The minimal fungicidal concentration of MB for 5 log10 CFU reduction (4.5 McFarland) was 80–160 μg/ml, whereas for RB it ranged between 320 and 640 μg/ml. For CUR, even the maximum studied concentration (1280 μg/ml) did not reach that inhibition. Incubation time had no effect in all experiments.

**Conclusions:**

Photodynamic therapy with RB, MB and CUR and white light is effective in killing *S. mutans* and *S. sanguis* strains, although MB and RB are more efficient than CUR. *C. albicans* required higher concentrations of all photosensitizers to obtain a fungicidal effect, being MB the most efficient and CUR ineffective.

## Background

The human oral cavity is colonized by a highly diverse community of bacteria [1]. Dental caries is a chronic, invasive disease involving demineralization of the tooth followed by destruction of the organic phase of the dentine [2] and it is the consequence of the interaction between oral microflora, diet, dentition and oral environment [3].

Streptococci are the main colonizers of oral surfaces and constitute 70 % of the cultivable bacteria existing in the human dental plaque [4]. In fact, *S. mutans* is the most prevalent microorganism of the plaque and the primary pathogenic agent responsible for caries disease [5], whereas *S. sanguis* is thought to play a benign, if not a beneficial, role in the oral cavity [6]. On the other hand, *C. albicans* is a commensal fungal species commonly colonizing human mucosal surfaces [7]. Falsetta et al. [8] hypothesize that *S. mutans*-*C. albicans* association may enhance *S. mutans* infection and modulate the development of hypervirulent biofilms on tooth surfaces, which will in turn influence the onset and severity of dental caries *in vivo*. For this reason, *S. mutans*, *S. sanguis* and *C. albicans* should be included in any study about human dental plaque microorganisms.

Photodynamic therapy (PDT) has been advocated as an alternative to antimicrobial agents to suppress subgingival species [9] due to the extensive and inappropriate use of antimicrobial agents which gradually led to the development of pervasive resistance [10]. Antimicrobial PDT (aPDT) is a technique that utilizes reactive oxygen species (ROS) produced by non-toxic dye or photosensitizer (PS) molecules in the presence of low intensity visible light to kill mammalian or microbial cells [11]. Due to this mechanism, It is hypothesized that bacteria will not be easily able to develop resistance to PDT [12].

More than 400 compounds with photosensitizing properties are known, including dyes, drugs, chemicals and many natural substances [13]. Methylene blue (MB), a well-known dye with high light absorption at 665 nm, is effective in aPDT, showing ability to kill not only Gram positive and Gram negative bacteria but also fungi [14–17]. Rose Bengal (RB) is a xanthene dye characterized by light absorption at wavelengths (λ) of 450–600 nm, used for the diagnosis of eye diseases [18]. From an antimicrobial point of view, RB has shown a good profile to photoinactivate microorganisms [19–22]. Curcumin (CUR) is an intensely yellow pigment, isolated from rhizomes of *Curcuma longa*, with a peak of light absorption at 430 nm [23]. Among its many biological activities are its anti-carcinogenic, antioxidant, antiinflammatory, antimicrobial properties and its hypoglycemic effects in humans [24, 25]. Some studies have shown its capacity to effectively photoinactivate in vitro *C. albicans* [26, 27].

There are many papers exploring the aPDT effect of different photosensitizers (PSs) in almost all kind of microbial species [28–31]. However, only few of them compare the efficacy of several photosensitizers on different microorganism [32].

The aim of this study was to compare the photoinactivation effect of three PSs, MB, RB and CUR, on S. *mutans*, *S. sanguis* and *C. albicans*.

## Results

### Photoinactivation of bacterial suspensions

Under the experimental conditions, PDT with MB, RB and CUR inhibited 6 log10 the growth of both strains of bacteria reaching a bactericidal effect. However, less concentration of RB than of the other PSs was needed to kill *Streptococcus* spp. Whereas this bactericidal effect was achieved for *S. mutans* with a concentration of RB as low as 0.31–0.62 μg/ml, higher MB concentration (1.25–2.5 μg/ml) was needed to reach the same reduction. In the case of *S. sanguis*, the RB and MB concentrations needed to obtain the same bactericidal effect were quite similar to those used for *S.mutans* (0.62–1.25 μg/ml and 1.25–2.5 μg/ml, respectively). Much higher concentrations of CUR were necessary to obtain the same reduction either for *S. mutans* or *S. sanguis* (Table 1).

**Table 1:** Minimal range concentration to reduce 6 log10 of *S. mutans* and *S. sanguis* and 5 log10 of *C. albicans*

Pre-irradiation Incubation time (h)	MB	RB	CUR
*S. mutans* ATCC 35668			
<1 min	1.25–2.5 μg/ml	0.31–0.62 μg/ml	80–160 μg/ml
1 h	0.62–1.25 μg/ml	0.15–0.31 μg/ml	160–320 μg/ml
3 h	0.62–1.25 μg/ml	0.31–0.62 μg/ml	160–320 μg/ml
*S. sanguis* ATCC 10556			
<1 min	1.25–2.5 μg/ml	0.62–1.25 μg/ml	160–320 μg/ml
1 h	0.31–0.62 μg/ml	0.15–0.31 μg/ml	40–80 μg/ml
3 h	0.15–0.31 μg/ml	0.15–0.31 μg/ml	40–80 μg/ml
*C. albicans* ATCC 1023			
<1 min	80–160 μg/ml	320–640 μg/ml	>1280 μg/ml
1 h	80–160 μg/ml	>1280 μg/ml	>1280 μg/ml
3 h	40–80 μg/ml	>1280 μg/ml	>1280 μg/ml

Irradiation with metal halide lamp, λ 420–700 nm, fluence 37 J.cm^−2^

Regarding the effect of the incubation time of *Streptococcus* cells with the PSs, one hour halved the minimal concentration of MB or RB necessary to attain 6 log10 reduction respect to an incubation time lower than 1 min (<1 min), especially for *S. sanguis* (Table 1). In the case of CUR, this effect was only observed for *S. sanguis*. Not significant additional benefit was achieved using 3 h of incubation (Table 1).

**Fig. 1:**
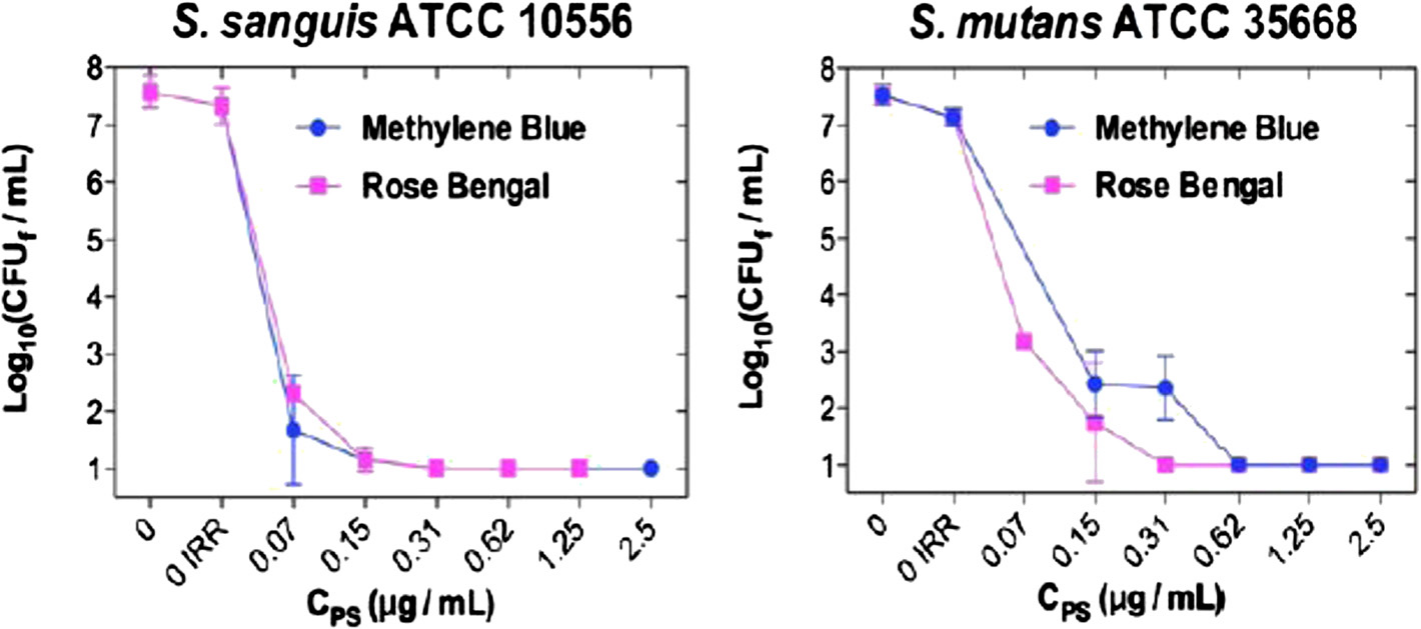
Photodynamic effect of MB and RB on *S. sanguis* and *S. mutans* depending on their concentration (Incubation time with the PS <1 min and irradiation using a metal halide lamp with a fluence of 37 J/cm^2^)

Comparing the photodynamic effect of MB for *S. mutans* and *S. sanguis* suspensions, using the optimal incubation for each PS, lower concentrations were needed to reach the bactericidal effect for *S. sanguis* than for *S. mutans* (Fig. 1). However, no differences were observed using RB as PS.

### Photoinactivation of *C. albicans*

**Fig. 2:**
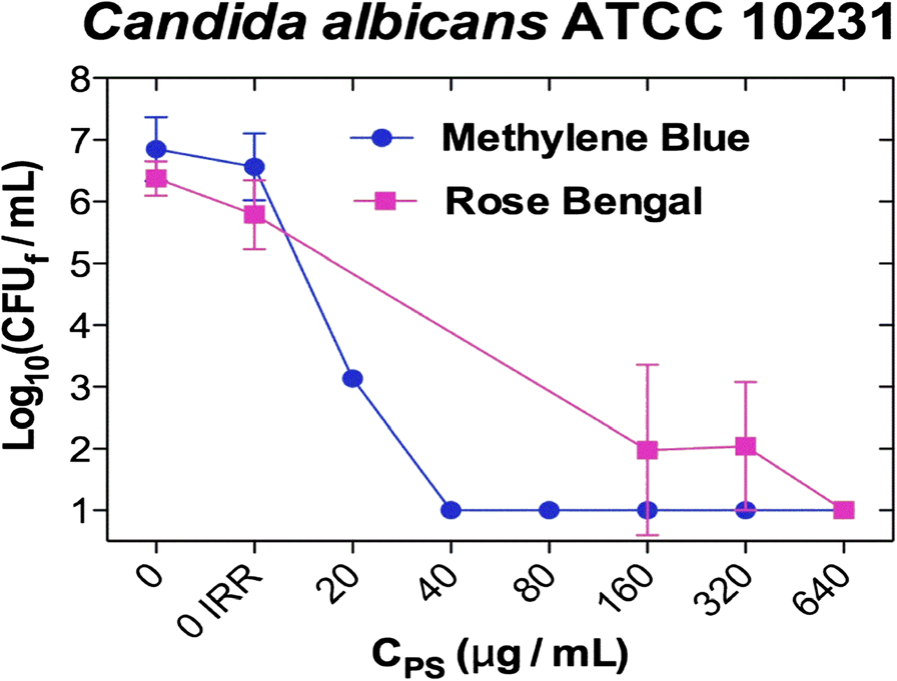
Photodynamic effect of MB and RB on *C. albicans* depending on their concentration (incubation time of 1 h to MB and <1 min to RB, and irradiation using a metal halide lamp whit a fluence of 37 J/cm^2^)

Table 1 shows the minimum fungicidal concentration (MFC) of each PS starting from *C. albicans* 4.5 McFarland. Under the experimental conditions, PDT with MB, RB but not with CUR inhibited 5 log 10 the growth of *C. albicans*, being the needed concentrations of MB smaller than the RB ones (Fig. 2). An increase in the incubation time with the PS was only beneficial for MB, because 3 h halved the concentration needed to reach a 5 log10 reduction in *C. albicans* respect to shorter times (Table 1).

## Discussion

Dental caries may be a disease well suited to PDT [2]. Our investigation showed that PDT using MB or RB and a white lamp can kill cariogenic microorganisms, such as *S. mutans*, *S. sanguis* and *C. albicans*. In contrast, even though PDT with CUR reaches the same bactericidal effect, much higher concentrations were needed and it was not effective against yeasts.

PDT efficacy depends on the microorganism, the PS and the light used. According to our results, RB showed higher antimicrobial photodynamic effect for *Streptococcus* spp than the other PSs studied, whereas MB was better for *Candida* spp. CUR always showed the lowest antimicrobial activity; this could be due to the fact that the higher peaks of the spectrum emission of the lamp correspond better to the absorptium spectra of RB and MB than CUR. Table 2 shows that compared with previous studies using aPDT with MB, RB and CUR for *S. mutans*, our parameters seem to be more efficient, especially considering the percentage of bacterial growth inhibition of 99.9999 %. Regarding the antimicrobial effect of MB-PDT on the viability of *S. mutans*, Araujo et al. [33] needed 25 μg/ml MB to reach 73 % inhibition. Nevertheless, our results are not completely comparable because they used red light, which corresponds with the maximum spectrum absorbance of MB. Regarding RB, studies carried out by Costa et al. [34] show that the concentration needed to attain 6.86 log10 CFU/mL reduction of *S. mutans* was 2.02 μg/ml, using a LED lamp with λ 440–460 nm and a fluence of 95 J/cm^2^. Comparing to our results they needed higher concentrations of PS to reach a similar effect, perhaps because the λ of their lamp was less convenient than ours to excite RB; another reason could be the use of distilled water as dissolvent, whereas they used phosphate-buffered saline because, based in the study of Nuñez et al. [35], a significant difference in the same aPDT experiment can be promoted only by the use of differents dissolvents.

**Table 2:** Summary of the in vitro PDT studies using methylene blue, rose Bengal or curcumin on *S. mutans, S. sanguis and C. albicans*

	PS	Concentration	Inhibition	λ	Fluence
		(μg/ml)	(%)	(nm)	(J/cm^2^)
*S. mutans*					
Araújo et al. [33]	MB	25	73	ND	ND
Our study	MB	2.5	999.999	420–700	37
Costa et al. [34]	RB	2.02	999.999	440–460	95
Our study	RB	0,62	999.999	420–700	37
Araújo et al. [23]	CUR	1500	99.9	450	5.7
Paschoal et al. [44]	CUR	1473.5	60	450	72
Manoil et al. [45]	CUR	0.73	95 %	360–550	542
Our study	CUR	160	999.999	420–700	37
*S. sanguis*					
Chan et al. [37]	AM	100	99–100	632.8	21.2
Our study	AM	2.5	999.999	420–700	37
Pereira et al. [32]	RB	5	9.9	455	95
Our study	RB	0.62	999.999	420–700	37
Our study	CUR	1500	999.999	420–700	37
Mattiello et al. [46]	TB	200	84.32	660	10
*C. albicans*					
Souza et al. [15]	AM	100	99.9	660	39,5
Peloi et al. [14] 4	AM	22,5	95,14	663	6
Souza et al. [16]	AM	100	88,6	685	28
Our study	AM	160	99.999	420–700	37
Costa et al. [18]	RB	23	9.9	455	95
Demidova et al. [47]	RB	200	99,9999	525–555	80
Our study	RB	>1280	<99.999	420–700	37
Andrade et al. [26]	CUR	7,3	89.5	455	5,28
Dovigo et al. [48]	CUR	14,8	85	440–460	18
Our study	CUR	>2460	<99.999	420–700	37

*PS* photosentizer, *MB* methylene blue, *RB* rose bengal, *CUR* curcumin, *TB* toluidine blue ortho, *ND* no data

According to this study, CUR needs much higher concentration than RB and MB to photoinactivate bacteria. This result agrees with those obtained by Araujo et al. [23] who, using a concentration of 1500 μg/ml and blue LEDs lamp (λ 450 nm, fluence 5.7 J/cm^2^), reached 60 % inhibition of *S. mutans* in planktonic cultures. However, other studies obtained 95 % reduction of *S. mutans* instead of 6 logs using only 0.73 μg/ml CUR, which could be explained by the higher fluence used (72 J/cm^2^), the λ of the lamp (blue light) and the lower percentage of bactericidal activity obtained.

Few studies compare the efficacy of different PSs to photoinactivate oral microorganisms. Rolim et al. [36] showed that MB, toluidine blue ortho, malachite green, erythrosine, eosin and RB, using a red LEDs lamp for the former and a blue one for the later, were photoactive in vitro against *S. mutans,* but only toluidine blue reduced 99.9 % of the microorganism. In this study, they also find a bactericidal effect of RB on *S. mutans* without light. Nevertheless, in our study no antimicrobial effects were observed when the strains were exposed either to the dyes or the light source separately.

Considering that the cariogenic potential of *S. sanguis* is deemed low compared to that of the *S. mutans*, the number of reports using aPDT to kill *S. sanguis* is lower than those of *S. mutans*. Chan et al. [37] demonstrated that PDT with MB was able to obtain a reduction of 99–100 % on cultures of *S. sanguis*. However, they used higher concentrations (100 μg/ml) than we used but lower fluence (21.2 J/cm^2^) of a diode laser (665 nm). Pereira et al. [32] only obtained a 9.9 % inhibition with 5 μg/ml RB and they used a higher fluence. Therefore, our results show MB as the most efficient bactericidal PS for *S. sanguis*.

According to the present investigation, MB has better antifungal profile than RB and CUR. Other authors [14, 15, 38], show that MB–aPDT is endowed with antifungal potential against *C. albicans,* whereas Costa et al. [18] show that RB only reach a 1,97 log10 reduction in *C. albicans*.

Comparing the three PSs, the present study shows that MB is the most effective PS on *C. albicans* while RB was slightly superior for *S. mutans*. Dental caries result from interactions among different cariogenic microorganisms, so using or combining different PSs could improve the efficacy of aPDT. In this sense, the use of a white light lamp that covers all the absorption spectrum of most PSs can efficiently excite them, making PDT easier to perform and avoiding the necessity of using a lamp for each PS. However, a light source with an emission spectrum that corresponds to the maximum absorption spectrum of each PS theoretically determines a higher efficacy [39]. Red light sources (630–700 nm) have been used extensively in PDT due to their relatively long wavelengths, which can effectively penetrate biological tissues and activates some of the most effective PSs, such as phenothiazines and porphyrins. Additionally, other studies have also shown that blue light (380–520 nm) is an attractive option for PDT, because blue light sources can be used in combination with many PSs, such as RB, eosin, erythrosine, and CUR to photoinactivate oral microorganisms [36]. For this reason, one limitation of our study is the use of the same lamp to photoactivate the three PSs, whose emission spectrum matches quite accurately with the maximum absorptium spectra of RB and MB but not with CUR. This could influence the bad results obtained with the later.

According to our data, the minimal bactericidal or fungicidal concentration was reduced in some experiments with a pre-irradiation incubation time of 1 h. However, considering that the increase in the concentration was only of one or two dilutions, the difficulty to maintain therapeutic concentrations of the PS in the high flow conditions within the oral cavity (due to saliva and/or gingival crevicular fluid) for a long period of time [40] do not support the use of incubation time in the clinical setting. Andrade et al. [26] using CUR and Rezusta et al. [41] with hypericin concluded that none incubation time enhance the photoinactivation of planktonic cultures of *C. albicans*. Additionally, although the adverse effects of blood and saliva could be avoided with the help of dental dams, not pre-incubation time is more comfortable for the patients and more efficient for doctors.

## Conclusions

The photodynamic efficacy of each PS varies according to the target microorganism. The combination of different PS and white light could be a promising approach to treat those infections caused by a combination of microorganisms, such as caries.

## Materials and methods

### Chemicals

Methylene Blue (MB) and Curcumin (CUR) were purchased from Sigma-Aldrich and Rose Bengal from Fluka. Sabouraud Dextrose Agar (SB) and Columbia Blood Agar (BA) were purchased from Oxoid.

### Microorganisms and growth conditions

*S. mutans* ATCC 35668, *S. sanguis* ATCC 10556 and *C. albicans* ATCC 10231 strains were obtained from the American Type Culture Collection (ATCC; Rockville, MD).

McFarland scale is recommended for performance of susceptibility testing by CLSI [42] and EUCAST [43].

The yeasts were grown aerobically overnight in SB medium at 35 °C. Stock inoculum suspensions were prepared in distilled water and adjusted to optical densities corresponding to 4.5 McFarland for five logs reduction assays. Cell viability was assessed counting the number of colony-forming units (CFU), developed on SB after an incubation period of 24 h at 35 °C.

*S. mutans and S. sanguis* were grown aerobically in BA medium at 35 °C for 48 h. Stock inoculum suspensions were prepared in distilled water and adjusted to optical densities corresponding to 0.5 McFarland for six logs reduction assays. Cell viability was assessed counting the number of CFU, developed on BA after an incubation period of 48 h for *S. mutans* and 24 h for *S. sanguis* at 35 °C.

### Photosensitizer solutions

Stock MB, RB and CUR solutions were prepared in distilled water and diluted either with bidistilled water to the desired concentration immediately prior to use. The concentrations used ranged from 0.1–12800 μg/ml. All solutions were prepared and handled under light-restricted conditions.

### Light source

**Fig. 3:**
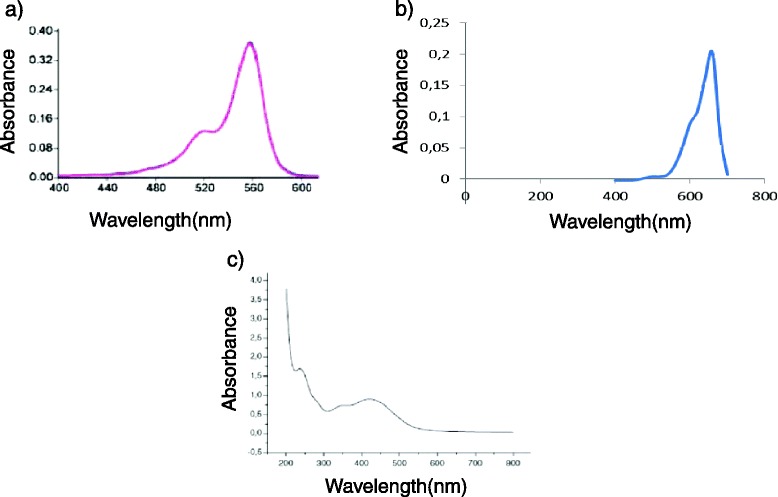
Spectrum absorption of rose Bengal **a**, methylene blue **b** and curcumin **c**

**Fig. 4:**
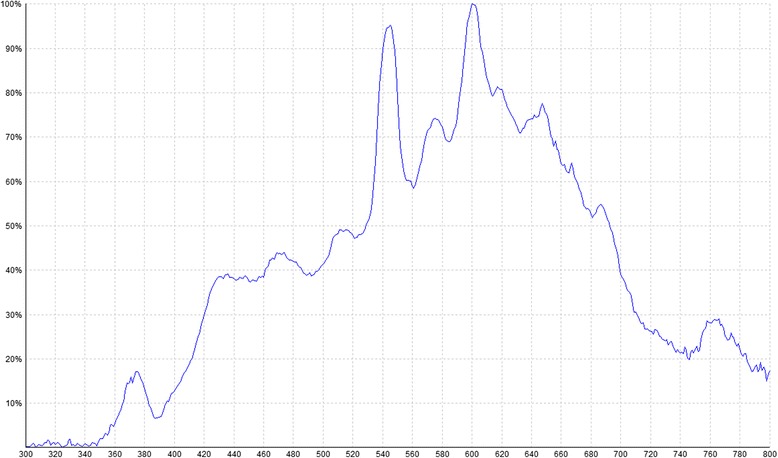
Relative emission curve of the metal halide lamp

In order to cover the spectrum absorption of the 3 PSs (Fig. 3), MB, RB and CUR (maximal absorption λ at 665, 557 and 430 nm respectively), we used a metal halide lamp emitting at 420–700 nm (Fig. 4) with an irradiance of 90 mW/cm^2^, being the specific irradiance at the maximal absorptium λ of each PS : 292 μW/cm^2^ at 557 nm, 300 μW/cm^2^ at 665 nm and 186 μW /cm^2^ at 430 nm.

Microorganisms suspensions with the different PSs prepared into a 96 wells microtiter plate, with a well diameter of 6 mm, were irradiated for 6 min and 51 s at a distance of 10 cm. The light beam diameter was 21 cm and the fluence used 37 J/cm^2^. The effective light received on the dishes results from integrating the power of the lamp for all the effective wavelengths for each PS.

### Photodynamic treatments of microorganisms

Suspensions with the desired McFarland value of every microorganism were prepared in bidistilled water. 90 μL of these initial suspensions was dropped into different wells of a microtiter plate and 10 μL of the different PS solutions were added. The final PS concentration in the experiments used ranged from 0.01–1280 μg/ml. The plates were then maintained in the dark for different periods of time (<1 min, 1 and 3 h) to evaluate the influence of contact time with the PS on the outcome of the photodynamic treatments. Afterwards, microorganisms were subjected to illumination (37 J/cm^2^).

Fungal and bacterial cultures grown under the same conditions with and without PS, either kept in the dark or illuminated, served as controls.

After photodynamic treatments, samples and controls were incubated at 35 °C for 24 h, in case of *C. albicans* and *S. sanguis* experiments, and for 48 h in case of *S. mutans*. The antimicrobial effect was determined by counting the number of CFU per millilitre.

A criterion of 5 log10 unit decrease from the starting inoculum was adopted to define fungicidal activity, and a more stringent criterion of 6 log10 unit for bactericidal activity, due to the differences in cell size and mass between *Candida* spp and *Streptococcus* spp (the cell concentration of *C. albicans* used was 10 times lower (>10^5^) than that used for the two bacterial species (>10^6^)).

All experiments were carried out at least five times.

## Abbreviations

MB: Methylene blue
RB: Rose bengal
CUR: Curcumin
CFU: Colony forming units
PDT: Photodynamic therapy
aPDT: Antimicrobial photodynamic therapy
PS: Photosensitizer
SB: Sabouraud dextrose agar
BA: Columbia blood agar

## References

[1] Vahabi S, Fekrazad R, Ayremlou S, Taheri S, Zangeneh N (2011). The effect of antimicrobial photodynamic therapy with radachlorin and toluidine blue on *Streptococcus mutans*: an in vitro study. J Dent (Tehran).

[2] Lima JP, de Melo MA S, Borges FM, Teixeira AH, Steiner-Oliveira C, Nobre Dos Santos M (2009). Evaluation of the antimicrobial effect of photodynamic antimicrobial therapy in an in situ model of dentine caries. Eur J Oral Sci.

[3] Marsh PD (1999). Microbiologic aspects of dental plaque and dental caries. Dent Clin North Am.

[4] Jenkinson HF (1994). Adherence and accumulation of oral streptococci. Trends Microbiol.

[5] Orasmo EMW, Otani C, Khouri S (2013). In vitro AFM evaluation of *Streptococcus mutans* membrane exposed to two mouthwashes. JAPS.

[6] Caufield PW, Dasanayake AP, Li Y, Pan Y, Hsu J, Hardin JM (2000). Natural history of *Streptococcus sanguinis* in the oral cavity of infants: evidence for a discrete window of infectivity. Infect Immun.

[7] Metwalli KH, Khan SA, Krom BP, Jabra-Rizk MA (2013). *Streptococcus mutans, Candida albicans*, and the human mouth: a sticky situation. PLoS Pathog.

[8] Falsetta ML, Klein MI, Colonne PM, Scott-Anne K, Gregoire S, Pai CH (2014). Symbiotic relationship between *Streptococcus mutans* and *Candida albicans* synergizes virulence of plaque biofilms *in vivo*. Infect Immun.

[9] Fontana CR, Abernethy AD, Som S, Ruggiero K, Doucette S, Marcantonio RC (2009). The antibacterial effect of photodynamic therapy in dental plaque-derived biofilms. J Periodontal Res.

[10] Ball AR, Tego GP (2012). Emerging antimicrobial drug-discovery strategies: an evolving necessity.

[11] Kharkwal GB, Sharma SK, Huang YY, Dai T, Hamblin MR (2011). Photodynamic therapy for infections: clinical applications. Lasers Surg Med.

[12] Zanin IC, Gonçalves RB, Junior AB, Hope CK, Pratten J (2005). Susceptibility of *Streptococcus mutans* biofilms to photodynamic therapy: an in vitro study. J Antimicrob Chemother.

[13] Meisel P, Kocher T (2005). Photodynamic therapy for periodontal diseases: state of the art. J Photochem Photobiol B.

[14] Peloi LS, Soares RR, Biondo CE, Souza VR, Hioka N, Kimura E (2008). Photodynamic effect of light-emitting diode light on cell growth inhibition induced by methylene blue. J Biosci.

[15] Souza RC, Junqueira JC, Rossoni RD, Pereira CA, Munin E, Jorge AO (2010). Comparison of the photodynamic fungicidal efficacy of methylene blue, toluidine blue, malachite green and low-power laser irradiation alone against *Candida albicans*. Lasers Med Sci.

[16] de Souza SC, Junqueira JC, Balducci I, Koga-Ito CY, Munin E, Jorge AO (2006). Photosensitization of different *Candida* species by low power laser light. J Photochem Photobiol B.

[17] Munin E, Giroldo LM, Alves LP, Costa MS (2007). Study of germ tube formation by *Candida albicans* after photodynamic antimicrobial chemotherapy (PACT). J Photochem Photobiol B.

[18] Costa AC, Rasteiro VM, Pereira CA, Rossoni RD, Junqueira JC, Jorge AO (2012). The effects of rose bengal- and erythrosine-mediated photodynamic therapy on *Candida albicans*. Mycoses.

[19] Chui C, Aoki A, Takeuchi Y, Sasaki Y, Hiratsuka K, Abiko Y (2013). Antimicrobial effect of photodynamic therapy using high-power blue light-emitting diode and red-dye agent on *Porphyromonas gingivalis*. J Periodontal Res.

[20] Bolean M, Paulino TP, Thedei G, Ciancaglini P (2010). Photodynamic therapy with rose bengal induces GroEL expression in *Streptococcus mutans*. Photomed Laser Surg.

[21] Freire F, Costa AC, Pereira CA, Beltrame Junior M, Junqueira JC, Jorge AO (2014). Comparison of the effect of rose bengal- and eosin Y-mediated photodynamic inactivation on planktonic cells and biofilms of *Candida albicans*. Lasers Med Sci.

[22] Guo Y, Rogelj S, Zhang P (2010). Rose Bengal-decorated silica nanoparticles as photosensitizers for inactivation of gram-positive bacteria. Nanotechnology.

[23] Araújo NC, Fontana CR, Bagnato VS, Gerbi ME (2012). Photodynamic effects of curcumin against cariogenic pathogens. Photomed Laser Surg.

[24] Wang D, Hu J, Lv L, Xia X, Liu J, Li X (2013). Enhanced inhibitory effect of curcumin via reactive oxygen species generation in human nasopharyngeal carcinoma cells following purple-light irradiation. Oncol Lett.

[25] Kunnumakkara AB, Anand P, Aggarwal BB (2008). Curcumin inhibits proliferation, invasion, angiogenesis and metastasis of different cancers through interaction with multiple cell signaling proteins. Cancer Lett.

[26] Andrade MC, Ribeiro AP, Dovigo LN, Brunetti IL, Giampaolo ET, Bagnato VS (2013). Effect of different pre-irradiation times on curcumin-mediated photodynamic therapy against planktonic cultures and biofilms of *Candida* spp. Arch Oral Biol.

[27] Garcia-Gomes AS, Curvelo JA, Soares RM, Ferreira-Pereira A (2012). Curcumin acts synergistically with fluconazole to sensitize a clinical isolate of *Candida albicans* showing a MDR phenotype. Med Mycol.

[28] Jackson Z, Meghji S, Macrobert A, Henderson B, Wilson M (1999). Killing of the yeast and hyphal forms of *Candida albicans* using a light-activated antimicrobial agent. Lasers Med Sci.

[29] Jori G, Fabris C, Soncin M, Ferro S, Coppellotti O, Dei D (2006). Photodynamic therapy in the treatment of microbial infections: basic principles and perspective applications. Lasers Surg Med.

[30] O’Riordan K, Akilov OE, Hasan T (2005). The potential for photodynamic therapy in the treatment of localized infections. Photodiagnosis Photodyn Ther.

[31] Lambrechts SA, Aalders MC, Van Marle J (2005). Mechanistic study of the photodynamic inactivation of *Candida albicans* by a cationic porphyrin. Antimicrob Agents Chemother.

[32] Pereira CA, Costa AC, Carreira CM, Junqueira JC, Jorge AO (2013). Photodynamic inactivation of *Streptococcus mutans* and *Streptococcus sanguinis* biofilms in vitro. Lasers Med Sci.

[33] Araújo PV, Teixeira KI, Lanza LD, Cortes ME, Poletto LT (2009). In vitro lethal photosensitization of *S. mutans* using methylene blue and toluidine blue O as photosensitizers. Acta Odontol Latinoam.

[34] Costa AC, Chibebe Junior J, Pereira CA, Machado AK, Beltrame Junior M, Junqueira JC (2010). Susceptibility of planktonic cultures of *Streptococcus mutans* to photodynamic therapy with a light-emitting diode. Braz Oral Res.

[35] Núñez SC, Garcez AS, Kato IT, Yoshimura TM, Gomes L, Baptista MS (2014). Effects of ionic strength on the antimicrobial photodynamic efficiency of methylene blue. Photochem Photobiol Sci.

[36] Rolim JP, De-Melo MA, Guedes SF, Albuquerque-Filho FB, De Souza JR, Nogueira NA (2012). The antimicrobial activity of photodynamic therapy against *Streptococcus mutans* using different photosensitizers. J Photochem Photobiol B.

[37] Chan Y, Lai CH (2003). Bactericidal effects of different laser wavelengths on periodontopathic germs in photodynamic therapy. Lasers Med Sci.

[38] Pupo YM, Gomes GM, Santos EB, Chaves L, Michel MD, Kozlowski VA (2011). Susceptibility of *Candida albicans* to photodynamic therapy using methylene blue and toluidine blue as photosensitizing dyes. Acta Odontol Latinoam.

[39] Calzavara-Pinton PG, Venturini M, Sala R (2007). Photodynamic therapy: update 2006. Part 1: Photochemistry and photobiology. J Eur Acad Dermatol Venereol.

[40] Wilson M (2004). Lethal photosensitisation of oral bacteria and its potential application in the photodynamic therapy of oral infections. Photochem Photobiol Sci.

[41] Rezusta A, López-Chicón P, Paz-Cristobal MP, Alemany-Ribes M, Royo-Díez D, Agut M (2012). In vitro fungicidal photodynamic effect of hypericin on *Candida* species. Photochem Photobiol.

[42] Clinical and Laboratory Standards Institute. Performance Standards for Antimicrobial Susceptibility Testing: 520 Q6. Twenty-Fourth Informational Supplement. CLSI document M 100-S24 (ISBN 1-56238-897-S (Print); ISBN 1-56238-898-3 (Electronic)). Wayne, PA: Clinical and Laboratory Standards Institute; 2014.

[43] The European Committee on Antimicrobial Susceptibility Testing. Breakpoint tables for interpretation of MICs and zone diameters. Version 4.0, 2014. http://www.eucast.org.

[44] Paschoal MA, Tonon CC, Spolidório DM, Bagnato VS, Giusti JS, Santos-Pinto L (2013). Photodynamic potential of curcumin and blue LED against *Streptococcus mutans* in a planktonic culture. Photodiagnosis Photodyn Ther.

[45] Manoil D, Filieri A, Gameiro C, Lange N, Schrenzel J, Wataha JC (2014). Flow cytometric assessment of *Streptococcus mutans* viability after exposure to blue light-activated curcumin. Photodiagnosis Photodyn Ther.

[46] Mattiello FD, Coelho AA, Martins OP, Mattiello RD, Ferrão Júnior JP (2011). In vitro effect of photodynamic therapy on *Aggregatibacter actinomycetemcomitans* and *Streptococcus sanguinis*. Braz Dent J.

[47] Demidova TN, Hamblin MR (2005). Effect of cell-photosensitizer binding and cell density on microbial photoinactivation. Antimicrob Agents Chemother.

[48] Dovigo LN, Pavarina AC, Carmello JC, Machado AL, Brunetti IL, Bagnato VS (2011). Susceptibility of clinical isolates of *Candida* to photodynamic effects of curcumin. Lasers Surg Med.

